# Supplementation of Crataegi fructus alleviates functional dyspepsia and restores gut microbiota in mice

**DOI:** 10.3389/fnut.2024.1385159

**Published:** 2024-04-02

**Authors:** Liyu Hao, Zeyue Yu, Jianhui Sun, Zongyuan Li, Jianliang Li, Yurong Deng, Hanhui Huang, Hairu Huo, Hongmei Li, Luqi Huang

**Affiliations:** ^1^School of Traditional Chinese Medicine, Shenyang Pharmaceutical University, Shenyang, China; ^2^Institute of Chinese Materia Medica, China Academy of Chinese Medical Sciences, Beijing, China; ^3^China Academy of Chinese Medical Sciences, Beijing, China

**Keywords:** Crataegi fructus, functional dyspepsia, gut microbiota, short-chain fatty acids, fecal microbiota transplantation

## Abstract

**Introduction:**

Functional dyspepsia (FD), also known as non-ulcerative dyspepsia, is a common digestive system disorder.

**Methods:**

In this study, an FD model was established using hunger and satiety disorders combined with an intraperitoneal injection of L-arginine. Indices used to evaluate the efficacy of hawthorn in FD mice include small intestinal propulsion rate, gastric residual rate, general condition, food intake, amount of drinking water, gastric histopathological examination, and serum nitric oxide (NO) and gastrin levels. Based on the intestinal flora and their metabolites, short-chain fatty acids (SCFAs), the mechanism of action of Crataegi Fructus (hawthorn) on FD was studied. The fecal microbiota transplantation test was used to verify whether hawthorn altered the structure of the intestinal flora.

**Results:**

The results showed that hawthorn improved FD by significantly reducing the gastric residual rate, increasing the intestinal propulsion rate, the intake of food and drinking water, and the levels of gastrointestinal hormones. Simultaneously, hawthorn elevated substance P and 5-hydroxytryptamine expression in the duodenum, reduced serum NO levels, and increased vasoactive intestinal peptide expression in the duodenum. Notably, hawthorn increased the abundance of beneficial bacteria and SCFA-producing bacteria in the intestines of FD mice, decreased the abundance of conditional pathogenic bacteria, and significantly increased the SCFA content in feces.

**Discussion:**

The mechanism by which hawthorn improves FD may be related to the regulation of intestinal flora structure and the production of SCFAs.

## 1 Introduction

Functional dyspepsia (FD) is a common type of functional gastrointestinal disorder. In clinical practice, it is characterized by upper abdominal pain, epigastric fullness, early satiety, belching, loss of appetite, nausea, and vomiting (1, 2). After excluding organic diseases, FD is one of the most common functional gastrointestinal diseases (3). Rome IV outlined the diagnostic criteria of FD as follows: dyspepsia occurring at least 3 days a week for more than 6 months in the past 3 months and not alleviated by defecation (4). According to existing studies, the etiology and mechanism of FD involve multiple factors. Abnormal gastrointestinal motility, visceral hypersensitivity, intestinal–brain axis disorders, immune system dysfunction, intestinal flora dysbiosis, and *Helicobacter pylori* (*H. pylori*) infection are all associated with FD (3, 5). Intestinal flora comprises all microbial communities living in the intestinal tract. The human digestive tract is the largest microbial pool in the body and is easily influenced by its ecological environment. With up to 10^14^ microbial entities and a gene sequence potential of 3.3 × 10^6^, which is approximately 150 times the total number of genes in the human body, it is also called “the second largest genome.” The intestinal flora plays an important role in human digestion and absorption, metabolism, energy supply, maintenance and construction of the intestinal mucosal barrier, and maintenance of intestinal microecological balance (6, 7). Changing the type and composition of the intestinal flora could offer a safe and effective approach to alleviate FD symptoms. Numerous cells and genes in the intestinal flora constitute a complex microecosystem *in vivo*. This system prevents the colonization of pathogens, acts as a biological barrier, participates in material metabolism, and regulates intestinal immunity (8). Studies have shown that FD is accompanied by changes in the composition of the intestinal flora (9). Recent studies have shown that an imbalance in the intestinal flora is an important factor in the pathogenesis of FD (10).

**Figure 1:**
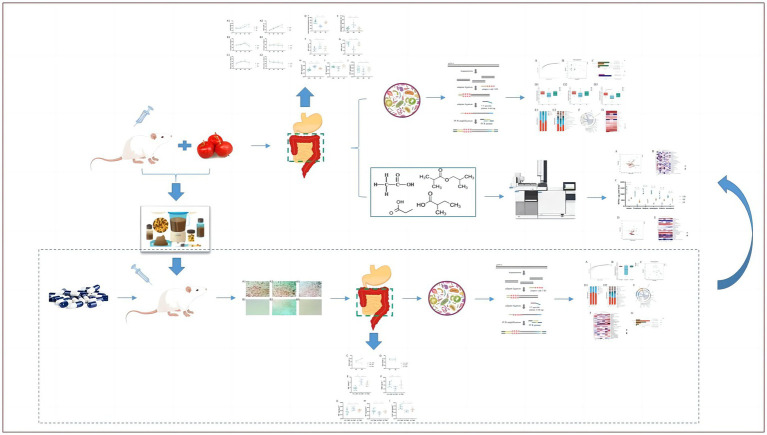
Brief flowchart of overall experimental design.

Crataegi fructus (hawthorn), the mature fruit of *Crataegus pinnatifida* Bunge, a deciduous tree species belonging to the family Rosaceae, is a medicinal and edible herb in China and is considered a popular dietary supplement globally (11). It is widely distributed in Asia, Europe, and North America. In Europe, it is made into canned fruits, jams, and jellies (12). Based on the literature, hawthorn fruits contain flavonoids, oligomeric proanthocyanidins, phenolic acids, triterpene acids, organic acids, and sterols. Flavonoids are the main active constituents of hawthorn extracts (13). Modern pharmacological studies have demonstrated that this extract has a therapeutic effect on dyspepsia (14). However, no conclusive experimental evidence indicating the exact efficacy and mechanism of hawthorn pairing in certain diseases is available. This study aimed to examine the therapeutic effects of hawthorn and explore its potential mechanism of action on microbial composition, function, and short-chain fatty acids (SCFAs) in FD mice. A brief flowchart of the experimental method is shown in Figure 1.

## 2 Materials and methods

### 2.1 Ethics statement

All animal procedures were performed in accordance with the Regulations of Experimental Animal Administration (Order No. 2, Approved by the State Council in 1988, third revision in 2017) issued by the State Committee of Science and Technology of the People’s Republic of China. This study was approved by the Institutional Animal Care and Use Committee (IACUC) of the Institute of Chinese Materia Medica, China Academy of Chinese Medical Sciences (Beijing, China), under code 2020B174.

### 2.2 Materials and sample preparation

Sliced and dried Crataegi fructus (Lot#2004001) were obtained from Anguo Changda Traditional Chinese Medicine Slices Co., Ltd. (Hebei, China). Dried Crataegi fructus (100 g) was decocted three times in 10 times the amount of pure water, each time for 1 h. The decoctions were combined and concentrated to 1 g/mL, then diluted to 0.3 g/mL before use.

### 2.3 Animals and experimental design

A total of 48 ICR (Institute of Cancer Research) mice (specific pathogen-free grade; body weight 18–22 g; equal male and female distribution) were purchased from Beijing Vital River Laboratory Animal Technology Co., Ltd. (Beijing, China, SCXK <jing> 2016-0006) and housed in the Animal Center (Beijing, China, SYXK<Jing> 2019-0003) at 20°C–26°C, 40%–70% humidity, under standard 12:12 h light–dark cycles. The mice were acclimatized for 3 days with free access to standard chow (Beijing Keao Xieli Feed Co., Ltd., Beijing, China, 21063213) and sterile water. Mice were randomly divided into control (CTL), functional dyspepsia (FD), and hawthorn (CF) groups by stratified randomization according to body weight. Each group consisted of 12 mice (six males and six females).

Hawthorn (6 g/kg) was administered intragastrically to mice in the CF group once daily for 30 days, while those in the CTL and FD groups were administered the same amount of purified water (20 mL kg^−1^) daily. Model induction began for all groups, except the CTL group, on the 14th day of administration. Two-day feeding, one-day fasting, and free drinking water cycles lasted for 2 weeks. From days 26–30, 1.5 g/kg L-Arg (Sigma-Aldrich, United States, SLCD5420) was injected intraperitoneally, whereas mice in the CTL group were injected with the same amount of normal saline (10 mL/kg).

The dietary dose of the aqueous hawthorn extract used in this study was 30 mg/kg, equivalent to approximately 12 g daily for a 70-kg adult, based on surface area dosage conversion factors. These doses of Crataegi fructus aqueous extract were acceptable for human consumption.

During the experiment, the mental state, stool shape, clustering, and activity of the mice in each group were observed and recorded daily.

### 2.4 Construction of FD pseudo-aseptic mouse model and intervention of bacterial solution

In total, 30 ICR mice (specific pathogen-free grade; body weight 18–22 g; equally distributed males and females) were randomly divided into control_FMT (CTL_FMT), functional dyspepsia_FMT (FD_FMT), and Crataegi fructus_FMT (CF_FMT) groups according to body weight. Each group consisted of 10 mice (5 males and 5 females). Mice received daily intragastric doses of ampicillin (0.39 g/kg), neomycin sulfate (0.26 g/kg), metronidazole (0.195 g/kg), and vancomycin (0.26 g/kg) for 7 days. On days 1 and 7, fresh feces were collected in sterile RNA-free tubes. The type and quantity of gut microbiota in the feces were detected using direct fecal smear and Gram staining, respectively. After successfully preparing the pseudo-aseptic mice, the corresponding fecal bacteria transplantation solution was intragastrically infused in the CTL_FMT, FD_FMT, and CF_FMT groups, with each mouse receiving 200 μL of the solution (the solution was prepared by mixing and filtering the feces of mice from the CTL, FD, and CF groups with aseptic saline at a ratio of 1:5 through a 0.20-mm pore size filter, followed by centrifugation at 7,000 r/min at 4°C for 5 min and resuspension of the lower sediment in aseptic saline at ratio 1:10). During this period, an FD model was established using L-Arg and hunger and satiety disorders.

### 2.5 Disease activity index assessment and sample collection

During the experiment, food intake and water consumption of mice were calculated every day.

On the 29th day after administration, all mice underwent a 16-h fast with access to water. On day 30, 1 h after administration, all mice were intragastrically infused with 5% charcoal powder solution. After 20 min, blood samples were obtained from the eyeballs. Subsequently, the gastric cardia and pylorus were ligated, and the small intestine was excised, starting from the gastric pylorus and ending at the ileocecal part. The propulsive length of the 5% charcoal powder solution and the total length of the small intestine were measured, and the small intestine propulsion rate was calculated.

$$\begin{array}{l} \mathrm{Small}\;\mathrm{intestinal}\;\mathrm{propulsion}\;\mathrm{rate}\;\left(\%\right)\\ =\left(\begin{array}{l} 5\%\mathrm{carbon}\;\mathrm{powder}\;\mathrm{solution}\;\mathrm{propulsion}\;\mathrm{length}\\ /\mathrm{total}\;\mathrm{small}\;\mathrm{intestinal}\;\mathrm{length} \end{array}\right)\times 100\% \end{array}$$

The stomach was weighed, and the total weight was recorded. The net weight of the stomach was determined after fully clearing the contents of the stomach, and the gastric residual rate was calculated.

$$\begin{array}{l} \mathrm{Intragastric}\;\mathrm{residual}\;\mathrm{rate}\;\left(\%\right)\\ =\left(\mathrm{total}\;\mathrm{gastric}\;\mathrm{weight}-net\;\mathrm{gastric}\;\mathrm{weight}\right)\\ /\mathrm{the}\;\mathrm{same}\;\mathrm{volume}\;\mathrm{of}\;5\%\mathrm{carbon}\;\mathrm{powder}\;\mathrm{solution}\times 100\% \end{array}$$

Peripheral blood samples were collected at the end of the experiment and stored at 4°C for 2 h before centrifugation at 3,000 r/min for 10 min. The serum was separated, and the Nitric Oxide (NO) Kit (Nanjing Jiancheng Bioengineering Institute, China, 20211105) and the Mouse Gastrin (GAS) ELISA Kit (Wuhan CUSABIO Co., China, Ltd., L210602439) were used to detect the NO and GAS contents in mouse serum. After blood collection, mice were sacrificed by cervical dislocation. Fecal samples were collected from the colon and stored at −80°C for further analysis. The duodenum of mice was used to prepare tissue homogenate, and 5% of the duodenum homogenate was used to determine the total protein by the BCA method (Nanjing Jiancheng Bioengineering Institute, China, 20210928). The 5-hydroxytryptamine (5-HT) ELISA Kit (Wuhan CUSABIO Co., Ltd., L211129838), the Mouse Substance P (SP) ELISA Kit (Wuhan CUSABIO Co., Ltd., L211012768), and the Mouse Vasoactive Intestinal Peptide (VIP) ELISA Kit (Wuhan CUSABIO Co., Ltd., L220113396) were used to determine the 5-HT, SP, and VIP levels in the duodenum.

### 2.6 Histopathological examination of gastric tissue

The gastric tissue was washed with 0.9% NaCl, the excess liquid was absorbed using filter paper, and the gastric mucosa was turned out and fixed in 10% formaldehyde. Subsequently, it was dehydrated in an ethanol gradient (50%, 70%, 95%, and 100% ethanol) and embedded in paraffin (*n* = 6 per group). The paraffin blocks were then sectioned at 3.5 μm, stained with hematoxylin and eosin (H&E), and histological changes in gastric tissues were analyzed.

### 2.7 Fecal SCFAs analysis using gas chromatography–mass spectrometry

The content of fecal SCFAs was determined using gas chromatography–mass spectrometry (GC–MS-QP2010 Ultra, Shimadzu, Japan) and quantified relative to acetic acid (Sigma-Aldrich, United States, BCCD6436), propionic acid (Sigma-Aldrich, United States, BCCF1438), isobutyric acid (Sigma-Aldrich, United States, CRACA252), butyric acid (Dr. Ehrenstorfer GmbH, Germany, 976513), isovaleric acid (Sigma-Aldrich, United States, BCCD7896), and isovaleric acid (Sigma-Aldrich, United States, BCCF0934).

After processing the samples were then subjected to GC–MS analysis using a Shimadzu capillary column WM-5MS (30 m × 0.25 mm × 0.25 μm), an injection volume of 0.5 μL at a split injection ratio of 30:1, and injection port, ion source, and transfer line temperatures of 250°C, 200°C, and 220°C, respectively. The temperature program was as follows: 27°C for 3 min, increased to 110°C at a rate of 4°C/ min, further increased to 250°C at a rate of 4°C/min, and maintained at 250°C for 1 min. Helium was used as a carrier gas at a flow rate of 1.0 mL/min. Mass spectrometry (MS) conditions were as follows: electron bombardment ion source, electron energy of 70 eV, and full scan (30–350 m/z).

### 2.8 Gut microbiota analysis

Microbial genomic DNA was extracted from fecal samples using the E.Z.N.A® soil DNA Kit (Omega Bio-tek, Norcross, GA, United States) according to the manufacturer’s instructions. The DNA extract was assessed using 1% agarose gel, and DNA concentration and purity were determined using a NanoDrop 2000 UV–vis spectrophotometer (Thermo Scientific, Wilmington, United States). The hypervariable region V3-V4 of the bacterial 16S rRNA gene was amplified with primer pairs 338F (5′-ACTCCTACGGGAGGCAGCAG-3′) and 806R (5′-GGACTACHVGGGTWTCTAAT-3′) using an ABI GeneAmp® 9700 PCR thermocycler (ABI, CA, United States). The PCR product was extracted from 2% agarose gel and purified using the AxyPrep DNA Gel Extraction Kit (Axygen Biosciences, Union City, CA, United States) according to the manufacturer’s instructions and quantified using a Quantus™ Fluorometer (Promega, United States).

Purified amplicons were pooled in equimolar amounts and subjected to paired-end sequencing on an Illumina MiSeq PE300 platform/NovaSeq PE250 platform (Illumina, San Diego, CA, United States), according to the standard protocols of Majorbio Bio-Pharm Technology Co. Ltd. (Shanghai, China).

### 2.9 Statistical analysis

Data are presented as mean ± SD for the indicated number of independently performed experiments.

Statistical analysis was performed using SPSS 23.0, and one-way analysis of variance (ANOVA) with Brown–Forsythe and Welch ANOVA tests were used to compare normally distributed data. Kruskal–Wallis and Dunn’s multiple comparison tests were used to compare abnormally distributed data.

## 3 Results

### 3.1 Hawthorn relieves symptoms of FD in mice

**Figure 2:**
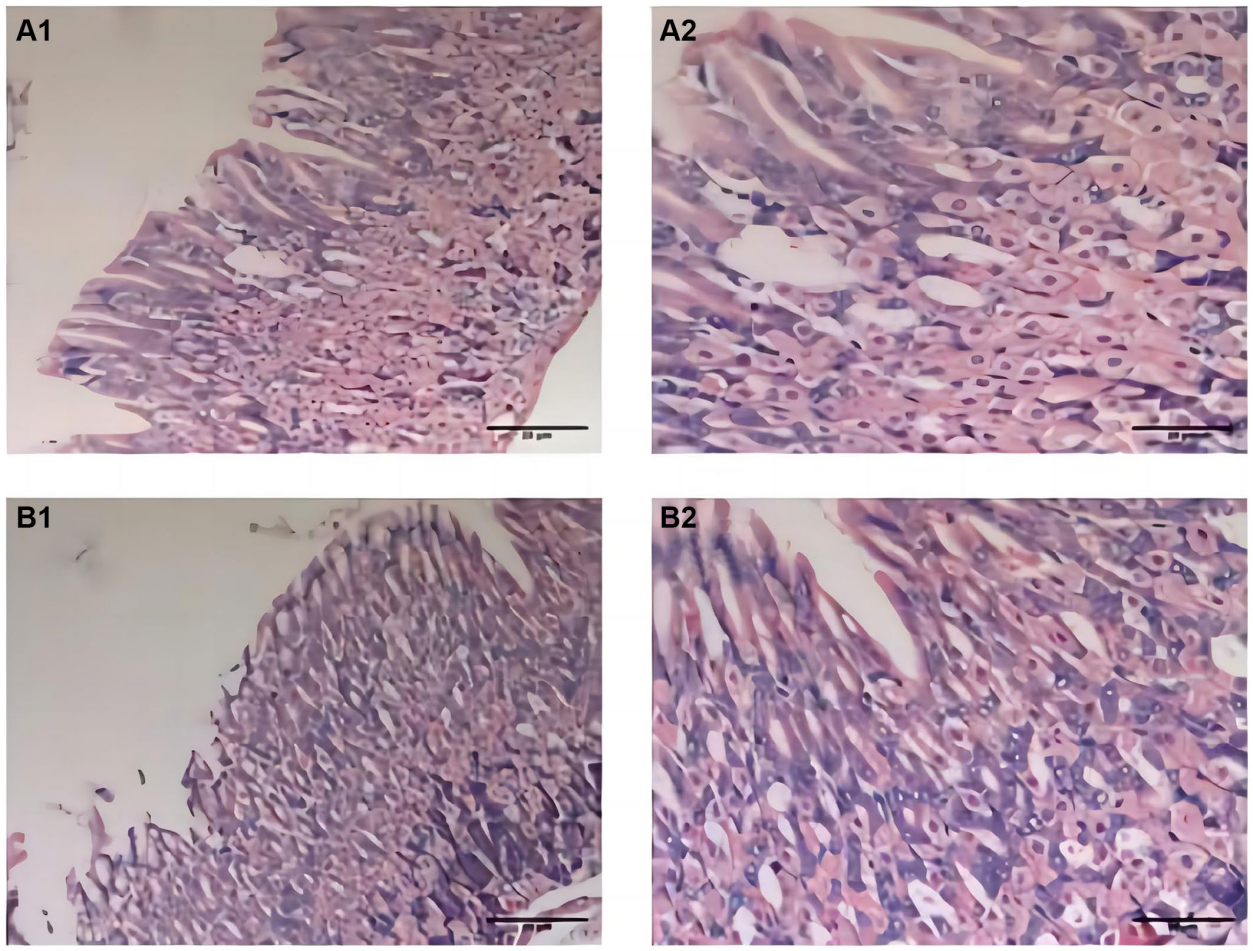
Pathological changes of gastric fundus histology mucosa by H&E staining (*n* = 6; scale bar, 20 μm) in the CTL **(A1–A2)** and model groups **(B1–B2)**. The gastric mucosa of mice in the CTL and model groups was intact and smooth; there were no exfoliation and inflammatory exudation; and the structure was normal.

During the experiment, the mice in the CTL group showed normal activity, fusiform stool, no sense of granules, and moderate humidity, while in the model group, the mice were sleepful, had reduced activity, loose stool mixed with granular sensation, poor degree of formation, and occasional anal prolapse. The pathological results showed that the gastric mucosa of mice in the CTL and model groups was intact and smooth, with no exfoliation or inflammatory exudation, and the structure was normal (Figure 2).

**Figure 3:**
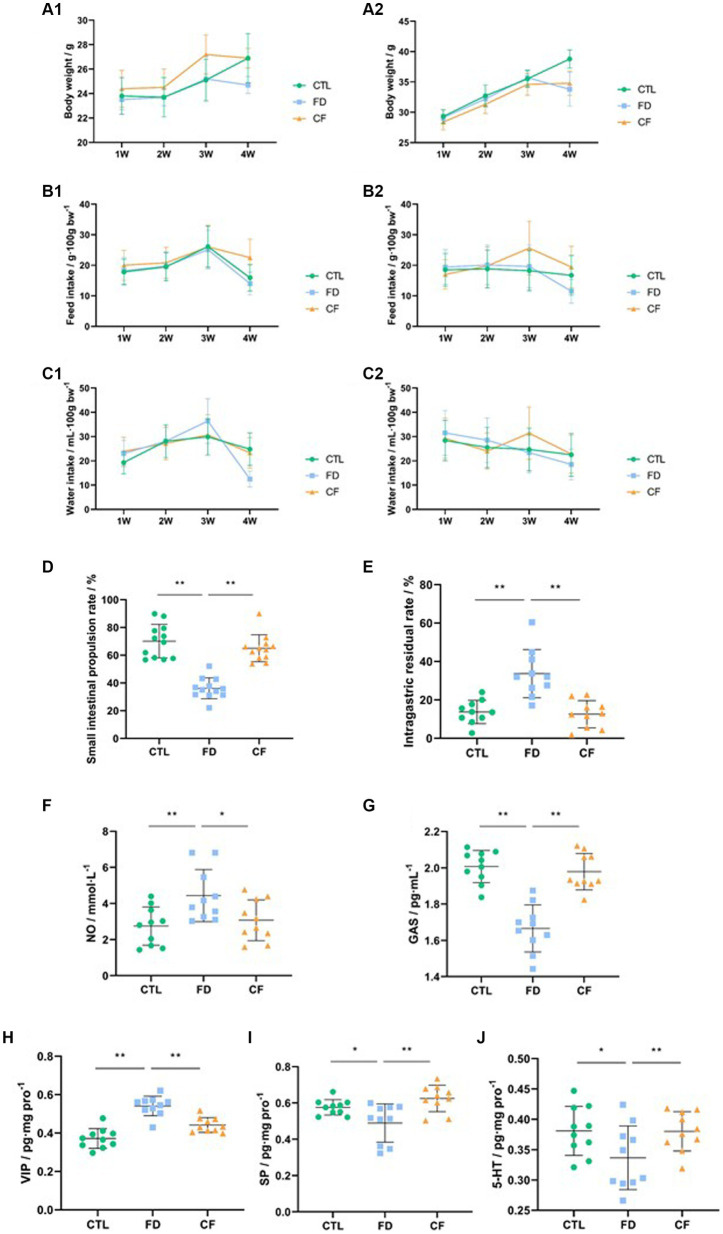
Hawthorn relieves symptoms of FD in mice. **(A1)** Body weight,♀; **(A2)** body weight,♂; **(B1)** food intake,♀; **(B2)** food intake,♂; **(C1)** water consumption,♀; **(C2)** water consumption,♂; **(D)** small intestinal propulsion rate; **(E)** intragastric residual rate; serum NO **(F)** and GAS **(G)** levels; VIP **(H)**, SP **(I)**, and 5-HT **(J)** levels in the duodenum. Data are presented as mean ± SD (*n* = 10). ^*^p < 0.05, ^**^p < 0.01.

The results indicated that mice in the model group experienced decreased body weight, reduced food intake, and lower water consumption compared to those in the CTL group. Conversely, mice in the *CF* group exhibited a slower weight loss trend and varying degrees of increased food intake and water consumption compared to mice in the FD group (Figures 3A1–C2).

Compared to the CTL group, the small intestinal propulsion rate significantly decreased (*p* < 0.01), the serum NO levels significantly increased (*p* < 0.05), the GAS levels significantly decreased (*p* < 0.05), the VIP levels in the duodenal homogenate significantly increased (*p* < 0.01), and the SP and 5-HT levels significantly decreased (*p* < 0.01) in the FD group. Compared to the FD group, the small intestinal propulsion rate significantly increased (*p* < 0.01), the serum NO levels significantly decreased (*p* < 0.05), the GAS levels increased significantly (*p* < 0.01), the VIP levels in the duodenal homogenate decreased significantly (*p* < 0.01), and the SP and 5-HT levels significantly increased (*p* < 0.05) in the CF group (Figures 3D–J).

### 3.2 Regulatory effect of hawthorn on intestinal flora in mice with FD

Species richness, diversity, and structural composition of the intestinal microflora in the CTL, FD, and CF groups were detected using 16S rDNA high-throughput sequencing technology, and the correlation between intestinal microflora and pharmacodynamic indices was analyzed.

**Figure 4:**
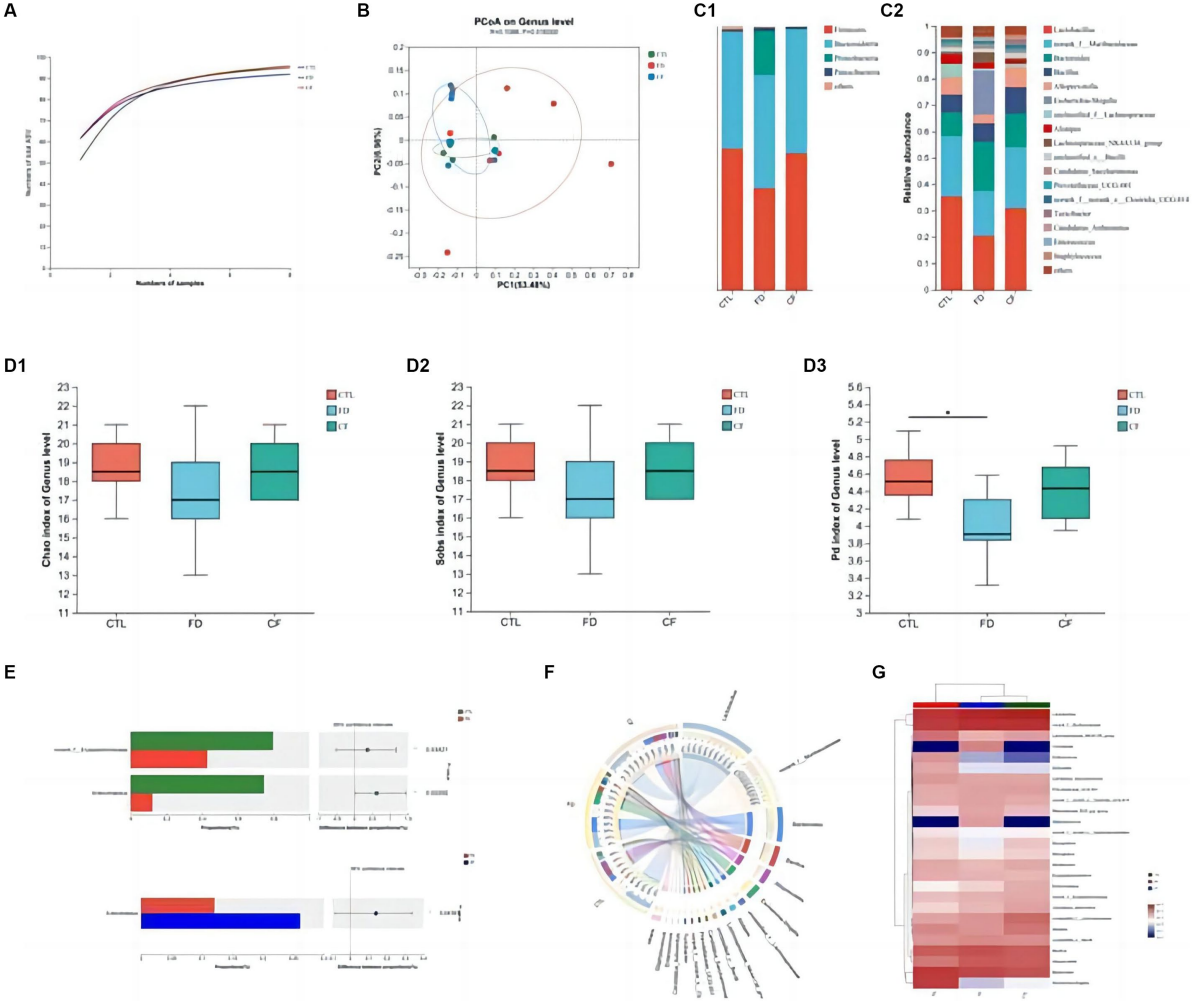
Regulatory effect of hawthorn on the intestinal flora in mice with FD. **(A)** Pan curve: Pan curve can be used to determine whether the sample size of this experiment is sufficient and reliable; **(B)** principal co-ordinates analysis of different groups; composition of gut microbiota at the phylum **(C1)** and genus **(C2)** levels; Chao estimator **(D1)**, Sobs estimator **(D2)**, and phylogenetic diversity **(D3)** of alpha diversity; **(E)** analysis between two groups; community Circos diagram **(F)** is a visual circle diagram that describes the corresponding relationship between samples and species; **(G)** heat map reflects the similarity and differences in community composition among different groups (or samples) at different classification levels through color changes and similarity levels. Data are presented as the mean ± SD (*n* = 8). ^*^p < 0.05, ^**^p < 0.01.

At the ASV classification level, as the sample size increased, the number of Pan species increased and then leveled off. This indicates that the sample size for each group of mice in this experiment was adequate, ensuring high credibility (Figure 4A).

Principal coordinate analysis revealed significant differences in the intestinal flora among the CTL, FD, and CF groups at the genus level, with *p* = 0.0160 and R = 0.1088. The high R-value indicates that the grouping has a substantial explanatory power for the observed differences, suggesting significant differences in the intestinal flora structure among the three groups. This demonstrates the high credibility of the test (Figure 4B).

At the phylum level, the FD group exhibited decreased Firmicutes abundance and increased Proteobacteria abundance compared to the CTL group. Conversely, the CF group showed increased Firmicutes abundance and decreased Proteobacteria abundance compared to the FD group (Figure 4C1). At the genus level, compared to the CTL group, the abundance of *Lactobacillus*, *Alloprevotella,* and *Alistipes* decreased. In contrast, the abundance of conditional pathogenic bacteria such as *Escherichia–Shigella* and *Enterococcus* increased. Compared with the FD group, the abundance of *Lactobacillus*, *Alloprevotella,* and *Alistipes* increased in varying degrees. In contrast, the abundance of pathogenic bacteria such as *Escherichia–Shigella* and *Enterococcus* decreased in the *CF* group (Figure 4C2).

Comparative analysis revealed that the abundances of *Lactobacillus* and *Dubosiella* in the intestinal flora of mice in the FD group were significantly lower than those in the CTL group (*p* < 0.05). Hawthorn-treated mice exhibited a more stable intestinal flora structure with increased richness and diversity compared to FD mice (Figures 4D1–D3). The abundance of probiotics and SCFA-producing bacteria increased, whereas that of conditional pathogenic bacteria decreased. Compared to the FD group, the abundance of *Lactobacillus* and *Dubosiella* in the intestinal flora of mice in the CF group decreased. This indicates that hawthorn can regulate the intestinal flora structure and restore disordered intestinal flora (Figures 4E–G).

**Figure 5:**
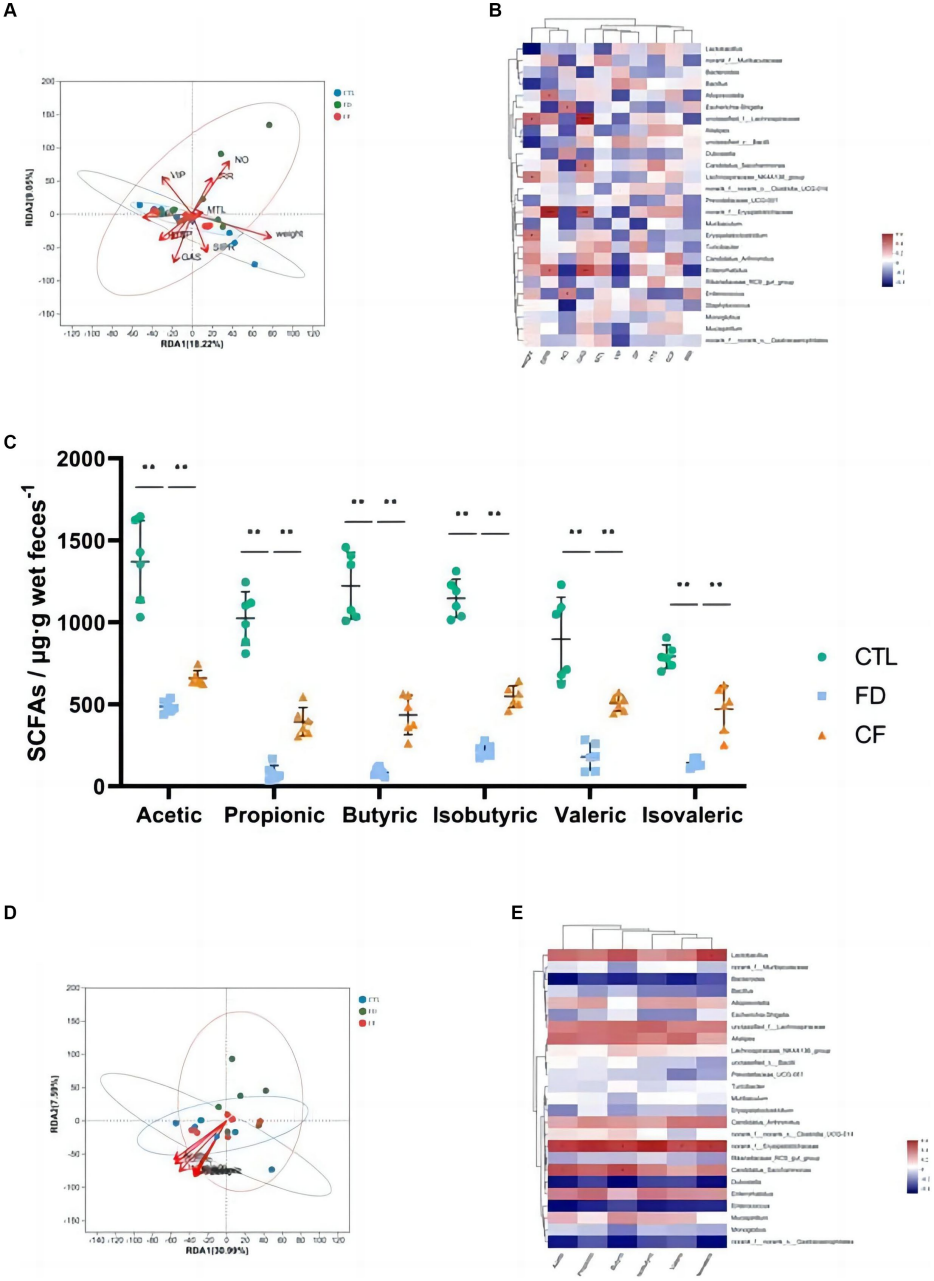
Correlation analysis among pharmacodynamic indicators, SCFAs, and gut microbiota. RDA/CCA analysis **(A)** and correlation heat map **(B)** of pharmacodynamic indicators and gut microbiota; **(C)** fecal SCFAs; RDA/CCA analysis **(D)**; and correlation heat map **(E)** of SCFAs and gut microbiota. Data are presented as mean ± SD (*n* = 10). ^*^p < 0.05, ^**^p < 0.01.

Using various physiological and biochemical indices as environmental factors, the relationship between intestinal flora species and environmental factors was analyzed using R-3.3.1. The results showed that body weight, small intestinal propulsion rate, intragastric residual rate, serum NO and GAS levels, and duodenal SP levels were closely related to the distribution of the intestinal flora species. Body weight, intragastric residual rate, and serum NO, GAS, and duodenal SP levels significantly correlated with intestinal flora species distribution (*p* < 0.05; Figure 5A).

The intestinal flora that significantly correlated with body weight (*p* < 0.05) included *Lactobacillus*, *Lachnospiraceae*, and *Alistipes*. Alloprevotella, Lactobacillus, and Erysipelotrichaceae significantly correlated with the small intestinal propulsion rate (*p* < 0.05). *Lachnospiraceae* and *Enterococcus* significantly correlated with the intragastric residual rate (*p* < 0.05). The intestinal flora that significantly correlated with the SP content in the duodenum (*p* < 0.05) include *Dubosiella*. *Alloprevotella* was the intestinal flora that significantly correlated with the VIP levels in the duodenum (*p* < 0.05). The intestinal flora that significantly correlated with 5-HT content in the duodenum (*p* < 0.05) was *Candidatus_Saccharimonas* (Figure 5B).

### 3.3 Analysis of SCFAs in feces and the relationship with gut microbiota

Hawthorn regulates the structure of the intestinal flora and affects its metabolic function and metabolite production. The results of this study revealed significant decreases in acetic, propionic, butyric, isobutyric, valeric, and isovaleric SCFAs in the FD group compared to the CTL group (*p* < 0.01). Conversely, compared to the FD group, the six types of SCFAs in the feces of mice in the CF group increased at different degrees (*p* < 0.01; Figure 5C). Correlation analysis revealed a correlation between SCFAs and intestinal flora, with six kinds of SCFAs significantly positively correlated with norank_f__*Erysipelotrichaceae* abundance (*p* < 0.05), and acetic and butyric levels were significantly positively correlated with Candidatus_*Saccharimonas* abundance (*p* < 0.05; Figures 5D–E).

### 3.4 Regulatory effect of fecal bacteria transplantation solution in mice with FD

Fecal flora from hawthorn-treated mice was transplanted into the FD model to mimic the pseudo-aseptic mice intestinal flora structure to verify whether hawthorn can improve FD by modifying the intestinal flora structure and its metabolites. Pseudo-aseptic mice were created by administering a combination of antibiotics (ampicillin, neomycin sulfate, metronidazole, and vancomycin) for 7 days (15).

**Figure 6:**
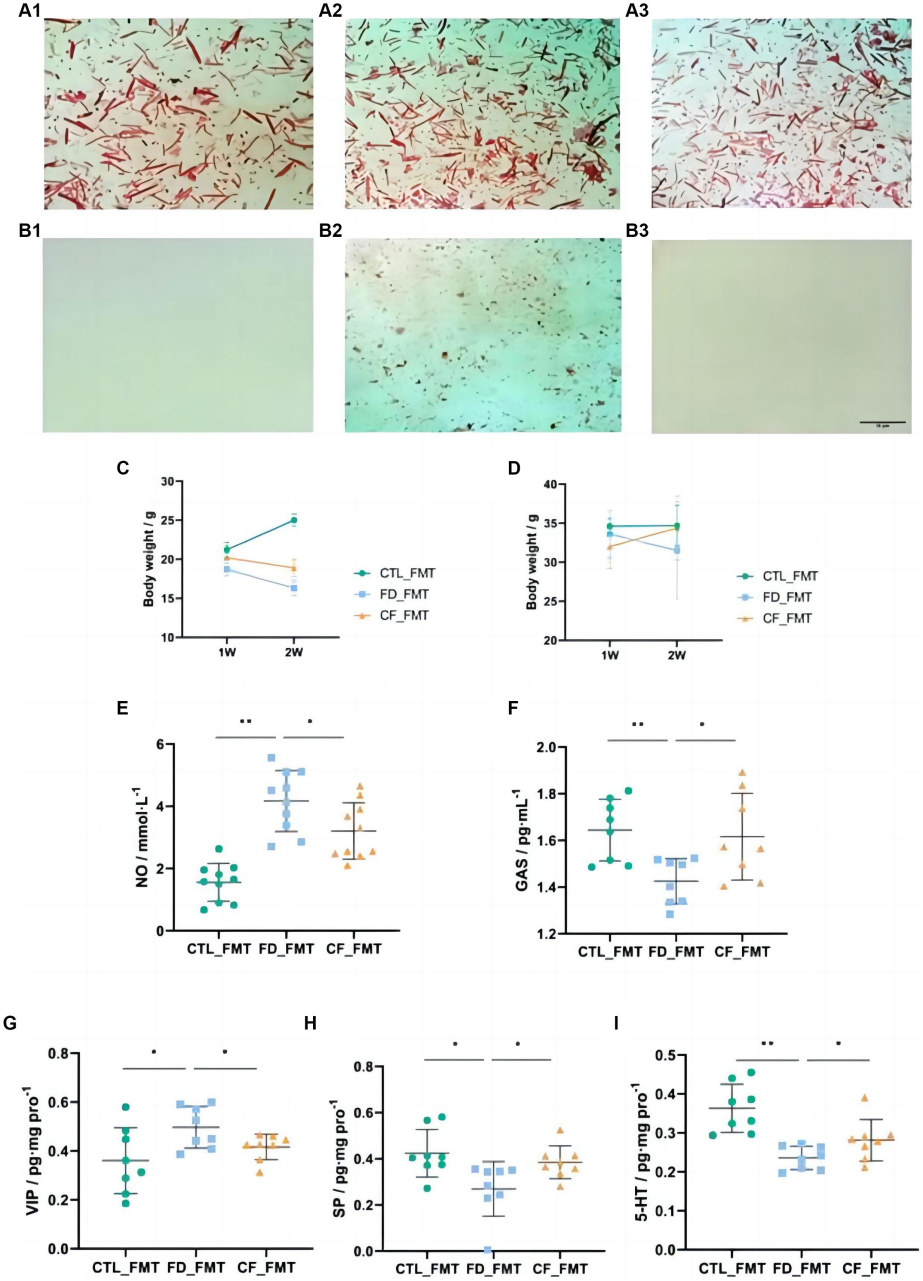
Effect of fecal bacteria transplantation solution on mice with FD. Fecal smears before **(A1–A3)** and after **(B1–B3)** antibiotics (Gram staining; scale bar, 10 μm); **(C)** body weight,♀; **(D)** body weight,♂; serum NO **(E)**; and GAS **(F)** levels; VIP **(G)**, SP **(H)**, and 5-HT **(I)** levels in the duodenum. Data are presented as mean ± SD (*n* = 8). ^*^p < 0.05, ^**^p < 0.01.

The fecal smears of mice before antibiotic administration contained more Gram-negative bacteria (blue) and Gram-positive bacteria (red), with almost no Gram-negative or Gram-positive bacteria after antibiotic administration, indicating the successful establishment of pseudo-aseptic mice (Figures 6A1–B3).

Compared with mice in the CTL_FMT group, mice in the FD_FMT group exhibited decreased body weight, the serum NO levels increased significantly (*p* < 0.01), and the GAS levels decreased significantly (*p* < 0.05), whereas in mice in the CF_FMT group the serum NO levels decreased significantly (*p* < 0.05) and the GAS levels increased significantly (*p* < 0.05) compared to the Model_FMT group (Figures 6C–F).

Compared to mice in the CTL_FMT group, the VIP levels in the duodenum of mice in the FD_FMT group increased significantly (*p* < 0.05), whereas the SP and 5-HT levels decreased significantly (*p* < 0.01). Compared to the Model_FMT group, the VIP levels in the homogenate of the 12 fingers in the CF_FMT group decreased significantly (*p* < 0.01), whereas the SP and 5-HT levels increased significantly (*p* < 0.05; Figures 6G–I).

### 3.5 Effect of fecal bacteria transplantation solution on the intestinal flora structure of mice with FD

**Figure 7:**
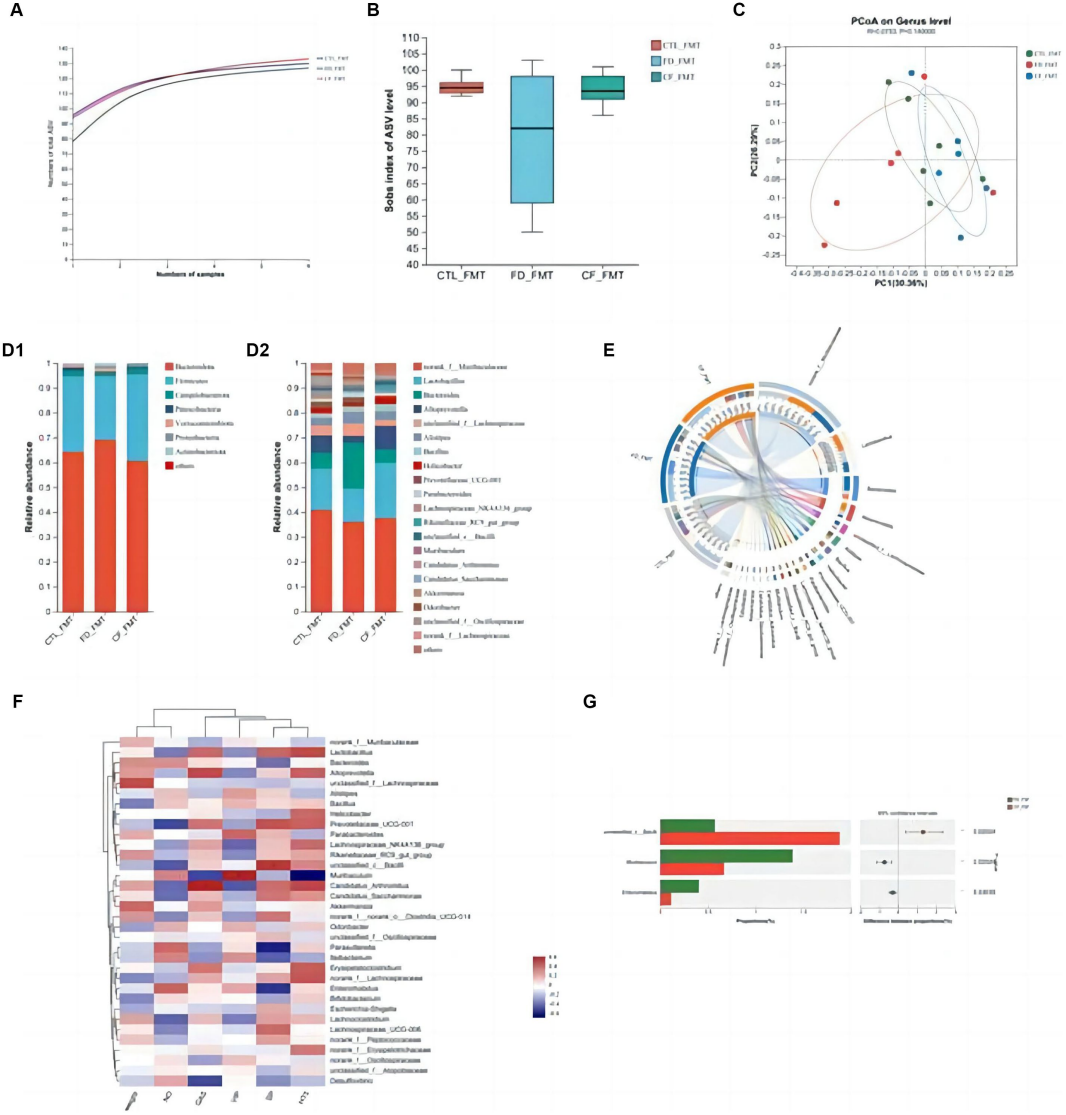
Effect of fecal bacteria transplantation solution of mice in the *CF* group on the intestinal flora structure of mice with FD. **(A)** Pan curve; Sobs estimator **(B)** of alpha diversity; **(C)** principal co-ordinates analysis of different groups; composition of gut microbiota at the phylum **(D1)**, genus **(D2)** levels, and community Circos diagram **(E)**; **(F)** heat map: reflects the similarity and differences in community composition among different groups (or samples) at different classification levels through color changes and similarity levels; **(G)** analysis between two groups. Data are presented as mean ± SD (*n* = 8). ^*^p < 0.05, ^**^p < 0.01.

At the ASV classification level, compared with the CTL_FMT group, intestinal flora richness, and diversity decreased in the FD_FMT group compared to the CTL_FMT group, whereas those in the CF_FMT group were higher than those in the FD_FMT group (Figures 7A,B).

Principal coordinate analysis revealed significant differences in the community composition among the CTL_FMT, FD_FMT, and CF_FMT groups at the genus level (Figure 7C). At the phylum level, Firmicutes and Bacteroidota were the dominant flora in all five groups. Compared to the CTL_FMT group, the abundance of Bacteroidetes decreased in the FD_FMT group. Compared to the FD_FMT group, the abundance of Bacteroidetes in the CF_FMT group increased. At the genus level, the abundance of *Lactobacillus* and *Alloprevotella* decreased, and that of *Enterococcus* increased in the FD_FMT group compared to the CTL_FMT group. Compared to the FD_FMT group, the abundance of *Lactobacillus* and *Alloprevotella* in the CF_FMT group increased compared to that in the FD_FMT group (Figures 7D–F).

Furthermore, *Lactobacillus* and *Alloprevotella* abundance decreased compared to that in the CTL_FMT group. The abundance of Bifidobacterium was significantly higher in the CF_FMT group than in the FD_FMT group (*p* < 0.05; Figure 7G). Generally, hawthorn fecal bacteria liquid can increase the abundance of beneficial bacteria and decrease the abundance of conditional pathogenic bacteria in the intestinal flora, consistent with prior findings from 16S rDNA high-throughput sequencing experiments.

## 4 Discussion

### 4.1 Hawthorn effectively alleviates FD in mice

In this study, an FD model was treated with an irregular diet combined with L-Arg administered intraperitoneally. This irregular diet is consistent with the etiology of FD. L-Arg is the only natural substrate for NO synthesis *in vivo*. L-Arg and oxygen can synthesize NO under the action of specific NOS, which inhibits gastrointestinal motility and causes symptoms of dyspepsia (16). Intraperitoneal administration of L-Arg increases Ca^2+^ in the cytoplasmic matrix, activates NOS, catalyzes L-Arg and oxygen to produce more NO, activates guanylate cyclase, catalyzes guanosine triphosphate to cyclic guanosine monophosphate, and causes gastrointestinal smooth muscle hyperpolarization and relaxation (2, 17).

The small intestinal propulsion rate, gastric residual rate, general condition, food and water intake, gastric histopathological examination, and serum NO and GAS levels were used as indices to evaluate the efficacy of hawthorn in FD mice. GAS, mainly produced by gastric antral G cells, stimulates gastric acid secretion, affects muscle cell contraction in various parts of the stomach, and promotes gastric mucosa repair (18). This study revealed that compared to the normal group, the model mice showed obvious dyspepsia symptoms such as reduced food intake, weight loss, and gastric emptying and small intestinal propulsion rates. The GAS levels showed changes in the visceral sensitivity of FD mice. Simultaneously, histopathological observation did not reveal any significant organic lesions. These results suggested that the FD mouse model prepared using this method showed changes in visceral sensitivity.

VIP is mainly located in nerve cells in the central nervous system and D cells in the gastrointestinal tract and plays an inhibitory role in relaxing smooth muscle and inhibiting gastrointestinal motility in the digestive system. SP and 5-HT are mainly distributed in intestinal chromaffin cells in the duodenum. SP promotes smooth muscle contraction (19). Approximately 95% of 5-HT is produced by chromaffin cells and binds to various receptors to transmit signals, which play an important role in regulating intestinal sensitivity. 5-HT exhibits two-way regulation, including binding with 5-hydroxytryptamine receptor 3 to promote gastrointestinal secretion while inhibiting gastrointestinal secretion via 5-hydroxytryptamine receptor 4. Hawthorn regulates gastrointestinal motility and alleviates FD by affecting the serum levels of GAS and NO, which may be related to the regulation of gastrointestinal motility and improvement of visceral hypersensitivity.

### 4.2 Regulatory effect of hawthorn on intestinal flora and SCFAs in mice with FD

Intestinal flora can decompose residual food in the host intestine, participate in the digestion and absorption of nutrients, and affect movement and secretion in the host intestine. The intestinal flora structure in FD mice was disordered, and its richness and diversity decreased. The abundance of probiotics and SCFA-producing bacteria decreased, whereas the abundance of conditional pathogenic bacteria increased. An increasing number of studies have shown that fecal bacterial transplantation is beneficial for the treatment of diseases such as irritable bowel syndrome and inflammatory bowel disease (20, 21).

The intestinal microflora decomposes food residues via anaerobic fermentation, and the metabolites produced are vitamins, bile acids, trimethylamine oxide, and SCFAs. SCFAs are also called volatile fatty acids because of their unstable structure and easy volatilization. They are closely related to the intestinal environment, and their species and content are affected by intestinal pH, composition and distribution of intestinal flora, glycolysis substrate, number and type of fermentation matrices, degradation rate, and host physiological state. SCFAs serve as nutrients for colonic epithelial cells, regulating the colon and cellular environment, cell proliferation, and gene expression. This study demonstrated that SCFA metabolites from intestinal flora impact host energy metabolism, highlighting their role as both flora metabolites and energy sources for colon cells, intricately related to the energy metabolism of the host.

The SCFAs in feces are mainly acetic, propionic, and butyric acids, accounting for 90–95%. Among them, acetic acid is produced by *Bifidobacterium* and *Clostridium*, propionic acid by *Clostridium*, and butyric acid by *Clostridium,* Firmicutes, and true rectal bacteria. Some studies have shown that the acetic acid content is the highest in the intestine, whereas the propionic and butyric acid contents are similar, with a ratio of 3:1:1 (22). After SCFAs are produced by intestinal microorganisms, approximately 95% of them are transported and absorbed across the membrane through monocarboxylate transporter 1 and sodium-coupled monocarboxylate transporter 1 on the surface of intestinal epithelial cells and participate in energy metabolism, and approximately 5% are metabolized by feces (23).

## 5 Conclusion

Hawthorn alleviated FD by significantly reducing the gastric residual rate, increasing the intestinal propulsion rate, increasing the intake of food and drinking water, increasing the levels of gastrointestinal hormones, upregulating SP and 5-HT expression in the duodenum, and downregulating the serum NO levels and VIP expression in the duodenum. Simultaneously, hawthorn increased the abundance of beneficial and SCFA-producing bacteria in the intestines of FD mice, decreased the abundance of conditional pathogenic bacteria, and significantly increased the SCFA content in feces. This was demonstrated through the FMT experiment. Generally, hawthorn (Crataegi fructus) can alleviate FD by affecting the intestinal flora structure and energy metabolism.

## Data availability statement

The datasets presented in this study can be found in online repositories. The names of the repository/repositories and accession number(s) can be found in the article/supplementary material.

## Ethics statement

The animal study was approved by the Institutional Animal Care and Use Committee (IACUC) of the Institute of Chinese Materia Medica, China Academy of Chinese Medical Sciences (Beijing, China), 2020B174. The study was conducted in accordance with the local legislation and institutional requirements.

## Author contributions

LHa: Writing – original draft. ZY: Writing – review & editing. JS: Writing – review & editing. ZL: Conceptualization, Data curation, Formal analysis, Funding acquisition, Investigation, Methodology, Project administration, Resources, Software, Supervision, Validation, Visualization, Writing – review & editing. JL: Writing – review & editing. YD: Writing – review & editing. HHua: Writing – review & editing. HHuo: Writing – review & editing. HL: Writing – review & editing. LHu: Writing – review & editing.
